# Automated Microinjection of Recombinant BCL-X into Mouse Zygotes Enhances Embryo Development

**DOI:** 10.1371/journal.pone.0021687

**Published:** 2011-07-20

**Authors:** Xinyu Liu, Roxanne Fernandes, Marina Gertsenstein, Alagammal Perumalsamy, Ingrid Lai, Maggie Chi, Kelle H. Moley, Ellen Greenblatt, Igor Jurisica, Robert F. Casper, Yu Sun, Andrea Jurisicova

**Affiliations:** 1 Department of Mechanical and Industrial Engineering and Institute of Biomaterials and Biomedical Engineering, University of Toronto, Toronto, Ontario, Canada; 2 Samuel Lunenfeld Research Institute, Mount Sinai Hospital, Toronto, Ontario, Canada; 3 Department of Obstetrics and Gynecology, Washington University in St. Louis, St. Louis, Missouri, United States of America; 4 Department of Obstetrics and Gynecology, University of Toronto, Toronto, Ontario, Canada; 5 Departments of Computer Science and Medical Biophysics, Ontario Cancer Institute and the Campbell Family Institute for Cancer Research, University Health Network, University of Toronto, Toronto, Ontario, Canada; VIB & Katholieke Universiteit Leuven, Belgium

## Abstract

Progression of fertilized mammalian oocytes through cleavage, blastocyst formation and implantation depends on successful implementation of the developmental program, which becomes established during oogenesis. The identification of ooplasmic factors, which are responsible for successful embryo development, is thus crucial in designing possible molecular therapies for infertility intervention. However, systematic evaluation of molecular targets has been hampered by the lack of techniques for efficient delivery of molecules into embryos. We have developed an automated robotic microinjection system for delivering cell impermeable compounds into preimplantation embryos with a high post-injection survival rate. In this paper, we report the performance of the system on microinjection of mouse embryos. Furthermore, using this system we provide the first evidence that recombinant BCL-XL (recBCL-XL) protein is effective in preventing early embryo arrest imposed by suboptimal culture environment. We demonstrate that microinjection of recBCL-XL protein into early-stage embryos repairs mitochondrial bioenergetics, prevents reactive oxygen species (ROS) accumulation, and enhances preimplantation embryo development. This approach may lead to a possible treatment option for patients with repeated in vitro fertilization (IVF) failure due to poor embryo quality.

## Introduction

According to the Centre for Disease Control, one in every eight North American couples seeks medical treatment for infertility. Embryo quality remains a strong determining factor for predicting the outcome of assisted reproductive technology (ART) [1]. Molecular defects responsible for failed preimplantation development are frequently attributed to poor oocyte quality of unknown etiology. Mathematical modeling of death rates in human preimplantation embryos has suggested that the factors predisposing an embryo to arrest are determined at or even before the zygote stage [2], [3]. The ability of the conceptus to pass through the transition from maternal to zygotic control *in vitro* has been proposed to be a function of the cytoplasmic components of the oocyte with minimal impact of the newly formed zygotic genome [4]. Thus, oocytes must possess cytoplasmic components which accumulate during oogenesis and support development through the blocking stage [5], and these components are lacking or non-functional in those embryos that arrest.

Ooplasm transfer experiments demonstrated that an unidentified ooplasmic factor(s) can prevent embryo arrest [6]. This pioneering work led to controversial clinical attempts to rescue human embryos with poor developmental potential by transferring ‘healthy’ donor ooplasm into recipient oocytes prone to abnormal development [7]. Such ooplasm transfers in humans were performed for patients with increased maternal age, repeated embryonic developmental failure or poor ovarian reserve, and have resulted in the birth of at least thirty children worldwide [8]. Unfortunately, transfer of ooplasm results in offspring carrying mitochondria from both the donor and recipient, thus creating mitochondrial heteroplasmy [9]. While benefits of this mitochondrial enrichment are clearly evident during the early developmental stages, mitochondrial heteroplasmy can have late physiological consequences [10]. Therefore, it is desirable to identify the molecular “culprits” responsible for suboptimal oocyte quality and devise molecular strategies for both fundamental research and treatment options.

Microinjection is a well established approach for introducing molecules into mammalian oocytes/embryos. High speed, high reproducibility, and high survival rate upon injection are critically important for testing the efficacy of potential molecular therapeutics. Due to the inherent difficulty of manipulating small-sized (∼100 µm), delicate mammalian embryos, conventional manual microinjection requires a long learning curve and suffers from low throughput.

The past decade has witnessed significant efforts to automate microinjection using robotic technologies [11], [12], [13], [14], [15], [16], [17], [18], and several robotic systems were demonstrated for assisting mouse embryo injection [11], [12], [18]. However, these robotic systems inherited the architecture directly from manual operation and only automated a few steps, while several difficult procedures (e.g., cell search, immobilization, and orientation) still must be conducted by a human operator with extensive training. Taking a different system architecture from manual operation and existing robotic systems, we have developed a novel robotic system that leverages motion control, computer vision microscopy, and micro device technology to achieve automated microinjection with high speed, reproducibility, and post-injection survival rate. This system employs microfabricated cell holding devices and vision-position-based control of multiple micropositioning devices to achieve easy sample immobilization, rapid cell orientation, and fast injection of mouse embryos. Technical aspects of the system were described previously [19], [20].

This paper reports the testing results of optimal system performance (e.g., injection speed and survival rate), based on the injection of a large number of mouse zygotes; and demonstrates a novel strategy for preventing early embryo arrest enabled by robotic microinjection of recombinant proteins into mouse zygotes. We provide the first quantitative evaluation of the efficacy of an anti-apoptotic recombinant protein BCL-XL (recBCL-XL) on rescuing early mouse embryos cultured in a suboptimal condition.

## Results

### Automated Microinjection of Mouse Zygotes

**Figure 1:**
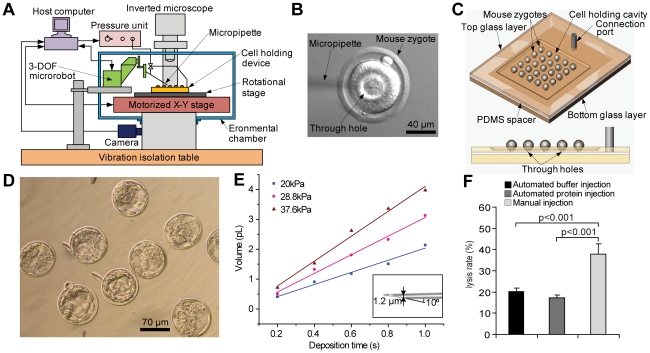
Automated robotic microinjection of mouse zygotes. (A) The robotic system employs a glass micro device to immobilize a large number of mouse zygotes into a regular pattern via fine vacuum and micrometer-sized through holes underneath cells. Based on precise position control and microscopy vision feedback, a three-degrees-of-freedom (3-DOF) micromanipulator, a motorized X-Y stage, and an in-house developed rotational stage are automatically controlled by a host computer to control an injection micropipette and position/orient the zygotes, respectively. An inverted microscope mounted with a digital camera is used to provide visual feedback and therefore, guide motions of the micropipette and zygotes to achieve automated microinjection. (B) A mouse zygote with the tip of a micropipette at the cytoplasmic center after material deposition. A droplet of mineral oil, which is easy to observe under a non-fluorescent microscope, was injected for visualization to verify the success of material deposition into the cytoplasmic center. (C) Schematic of the glass micro device for zygote immobilization. Vacuum is applied to each zygote for immobilization via micrometer-sized through holes. (D) Mouse zygotes robotically injected with PBS buffer are developed into blastocysts. (E) Calibration data of deposition volumes as a function of deposition time and pressure. Micropipettes with an opening of 1.2 µm were used, as shown in the inlet. (F) Automated robotic microinjection induced significantly lower lysis rates than manual injection (n = 400 for robotic protein and buffer injection; n = 229 for manual injection). Bars indicate mean ± s.e.m. Kruskal Wallis test followed by Dunn's post test was used for statistical analysis.

In contrast to conventional manual injection systems, our robotic system (Figure 1A) uses a microfabricated glass cell holding device (Figures 1C and S1A) to immobilize many mouse zygotes into a regular pattern (Figure S1C, Text S1, and Video S1). Switching from one cell to another for injection was greatly simplified and automatically performed via precise position control (Figures S2–S3, Text S1, and Video S2), dramatically enhancing the injection speed. A vision-based cell orientation control technique (Text S1) as well as an in-house developed motorized rotational stage (Figure 1A) was integrated into the robotic system for fast and automated cell orientation. A motorized micromanipulator (i.e., injection microrobot) was automatically controlled to inject mouse zygotes (Figure 1B and Video S2) in a high-speed manner with high repeatability.

During system development, 306 mouse zygotes were injected with PBS buffer by the robotic system. Through these trials, an injection speed of 200 µm/s and a retraction speed of 500 µm/s were experimentally determined to be optimal in terms of minimizing injection-induced cell lysis. In order to ensure viability of injected zygotes and investigate dose effects of the injected materials, deposition volume was accurately controlled (Figure 1E), achieved by fabricating injection micropipettes with a high consistency and precisely regulating the pressure unit output (Text S1). The resolution of material deposition volume was 1 femtoliter (fL).

To quantify system performance, the robotic system injected an additional 240 mouse zygotes with PBS buffer, demonstrating an average injection speed of 12 zygotes/min (vs. ∼2 zygotes/min in manual injection by highly skilled technicians, data provided by microinjection operators at the Toronto Center for Phenogenomics). Based on visual inspection right after injection, the robotic system achieved a low cell lysis rate (1.1%, Table 1). Developmental competence of microinjected embryos assessed by the rate of blastocyst formation (Table 1) *in vitro* after 96 hours in culture was 89±1.3% (mean ± s.e.m.), indicating minimal detrimental effect of robotic injection on embryo quality.

**Table 1:** Statistics of non-lysis and blastocyst formation rates of mouse embryos with PBS injection using the robotic system.

Experiments	1	2	3	4	5	6	7	Overall
Number of injected embryos	18	18	27	27	50	50	50	**240**
Number of surviving embryos (day 1.5)	18	18	26	27	50	49	49	**237**
Number of blastocysts (day 4.5)	16	15	24	23	46	45	44	**213**
Non-lysis rate (%)	100	100	96.3	100	100	98	98	**98.9±0.6 (mean±s.e.m.)**
Blastocyst formation (%)	88.9	83.3	92.3	85.2	92	91.8	89.8	**89±1.3 (mean±s.e.m.)**

The injected embryos were cultured in KSOM medium.

Due to the higher viscosity of the recombinant proteins (vs. PBS buffer), we next determined the need for increased size of microinjection pipettes. This modification increased the embryo lysis rates (20.3%; Figure 1F); however, the robotic system still provided significantly lower lysis rates than manual injection (37.7%; Figure 1F). There was no significant difference between the lysis rates of protein injection or buffer injection produced by robotic and manual microinjection. Thus, the robotic system has better performance in terms of cellular damage caused by microinjection. Automation enables users to operate the system without the long training needed for microinjection while achieving high injection consistency.

### Culture induced developmental arrest

**Figure 2:**
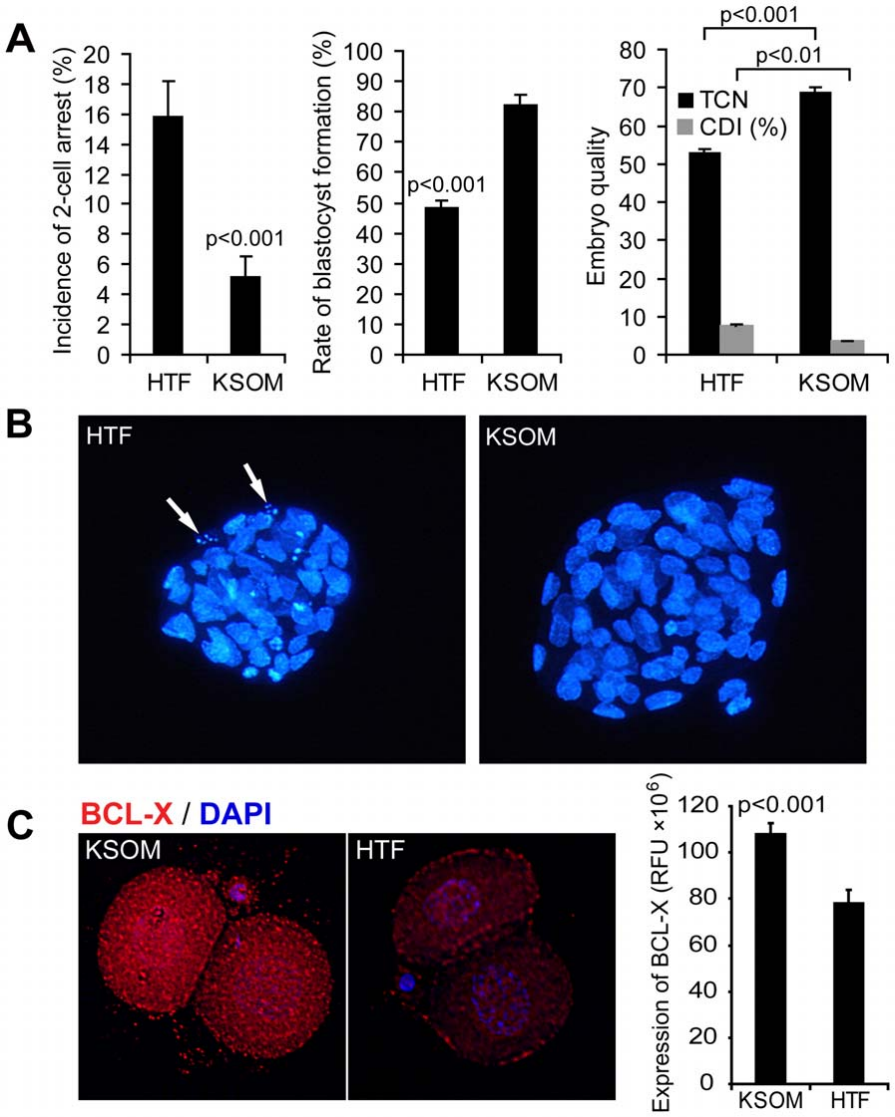
Impact of culture medium on developmental competence and BCL-X protein expression of mouse embryos. (A) HTF culture medium induces 2-cell arrest in a subset of embryos and compromises preimplantation embryo development when compared to KSOM medium (n = 273 embryos/medium). Rates of blastocyst formation at day 4.5 (∼96 hours in culture) as well as total cell number (TCN) are dramatically reduced, while cell death index (CDI) is elevated (n = 54 embryos for KSOM and n = 36 embryos for HTF). Bars indicate mean ± s.e.m. Mann Whitney U-test was used for pairwise comparison. (B) Poor quality of embryos is also reflected by nuclear staining (DAPI), showing smaller blastocysts with multiple apoptotic cells (arrows). (C) Expression of BCL-X protein is decreased in 2-cell embryos cultured for 24 hours in HTF medium. Significant reduction in fluorescent intensity (RFU), generated after immunocytochemical analysis for BCL-X was detected in embryos cultured in HTF (n = 10), when compared to KSOM cultured embryos (n = 9). Control embryos, exposed to no-specific IgG (n = 6), exhibited only very small amount of fluorescence, which was subtracted from the intensity generated by BCL-X antibody. Bars indicate mean ± s.e.m. Student's t-test was used for calculating significance of difference between KSOM and HTF groups.

To use the system to address interesting questions in developmental biology, we focused on devising strategies for overcoming early embryo arrest. Outbred colonies of mice often exhibit compromised preimplantation embryo development in suboptimal culture conditions [21]. We first determined that human tubal fluid (HTF) medium, often used by *in-vitro* fertilization (IVF) clinics in the past, delays mouse preimplantation embryo development *in vitro* (Figures 2A and 2B). The HTF culturing model well recapitulates developmental arrest and was used to study the impact of ooplasmic transfer in the mouse model [22]. We and others have previously reported that embryos of suboptimal quality often exhibit altered expression levels of genes known to regulate cell death [23], [24]. In addition, females lacking *Bcl-x* (officially called *Bcl2L1*) in their oocytes exhibit decreased breeding performance that could not be attributed to a defect in ovarian reserve [25]. In order to determine whether altered levels of Bcl-2 family members accompany embryo arrest in the mouse, we explored if BCL-X protein levels changed in 2-cell stage mouse embryos due to culture in HTF medium.

Immunocytochemistry revealed ∼25% reduction of BCL-X protein expression in the embryos that were cultured for 24 hours in HTF in comparison to potassium simplex optimization medium (KSOM) (Figure 2C), most commonly used for *in vitro* culture of murine embryos. These results suggest that the depletion of BCL-X may contribute to poor embryo survival in the suboptimal culture conditions and may be one of the cytoplasmic factors responsible for improved embryo development observed during ooplasmic rescue.

### Enhancing embryo development by automated microinjection of recBCL-XL (ΔTM)

**Figure 3:**
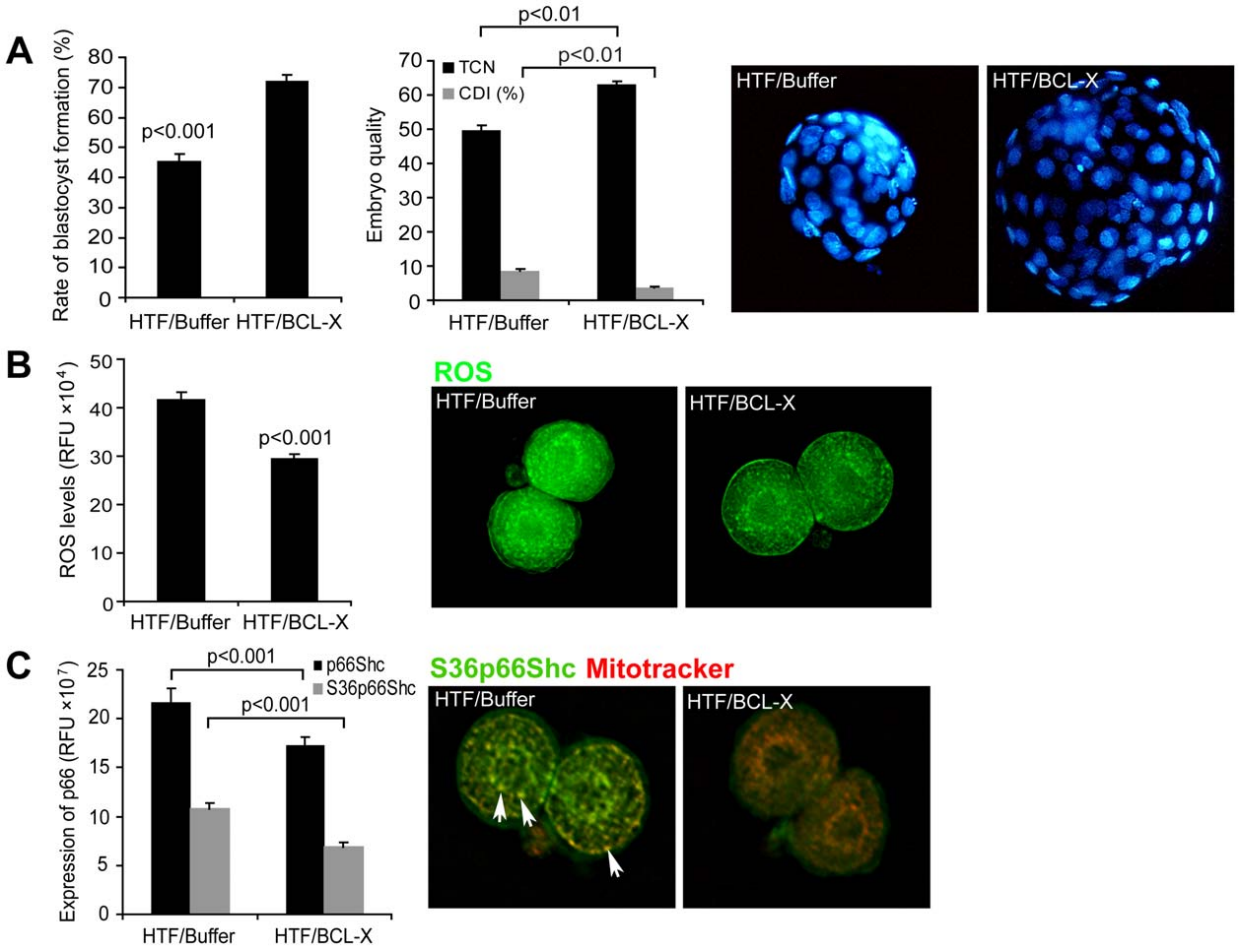
Impact of recBCL-XL (ΔTM) microinjection on early embryo development. (A) Ability of mouse zygotes to progress through the development and form blastocysts in suboptimal HTF medium were significantly increased upon microinjection of recBCL-XL (ΔTM) protein (n = 424) when compared to buffer injected embryos (n = 414). In addition, total cell number (TCN) per embryo was significantly increased and cell death index (CDI) was decreased (n = 71 for buffer injection; n = 110 for protein injection). Nuclear counterstaining (DAPI) images of blastocysts at day 4.5 reflect differences in embryo quality. Mann-Whitney U-test was used for pairwise comparison. (B) Reactive oxygen species (ROS) accumulation, determined by fluorescent measurement of DCHFDA probe fluoresce at 2-cell stage was determined 24 hours after microinjection of either buffer (n = 15) or recBCL-XL (ΔTM) protein (n = 15) and relative fluorescence units (RFU) were used to express fluorescent signal. Injection of recBCL-XL (ΔTM) significantly reduced the accumulation of ROS (student's t-test). (C) Immunocytochemical analysis of total p66SHC or phosphorylated p66SHC on Ser36 was decreased in embryos injected with recBcl-xL (ΔTM) (n = 15/antibody), when compared to buffer injected embryos (n = 15/antibody). In addition, we noticed that Ser10 p66SHC (green) localized to the mitochondria (Mitotracker red), with preferential clustering in subcortical and peri-nuclear regions (yellow overlap; arrows), but this was greatly reduced in recBCL-XL (ΔTM) microinjected embryos. Bars indicate mean ± s.e.m.

Thus, we next attempted to transiently supplement BCL-X levels by microinjecting recBCL-XL (ΔTM) [26] into zygotes and to examine their *in vitro* developmental potential under an adverse culture condition (HTF). Injection of recBCL-XL (ΔTM) protein significantly improved preimplantation embryo development, when compared to buffer-injected, HTF-cultured embryos (p<0.001; Figure 3A). Rates of blastocyst formation, total cell number (TCN) and cell death index (CDI), which all reflect embryo quality, were restored by recBCL-XL (ΔTM) microinjection to levels comparable with embryos cultured in KSOM medium (Figure 3A and Figure 2). As a negative control, we also injected zygotes with BSA dissolved in microinjection buffer, and this did not significantly improve developmental rates (47%; n = 66) or embryo quality (TCN: 64±5.5%, CDI: 4.2±0.6; n = 13). These results show that microinjection of the recBCL-XL (ΔTM) protein is capable of restoring developmental competence and improving quality of embryos facing conditions of stress. Furthermore, there was no significant difference in studied outcomes if protein was delivered into the cytoplasm or pronucleus (Figure S4). As robotic microinjection resulted in lower lysis rates and a higher degree of consistency, all experiments described below were performed with robotic recBCL-XL (ΔTM) delivery into the cytoplasm.

Gametes and early mammalian embryos are susceptible to damage caused by excessive reactive oxygen species (reviewed in [27]). While reactive oxygen species (ROS) are key signaling molecules mediating basic cellular functions such as proliferation, differentiation and programmed death, excessive ROS production has been implicated in DNA damage, ATP depletion and permanent embryo arrest similar to that of cellular senescence [28]. As BCL-X has been previously shown to have antioxidant activities [29], we explored if BCL-X can increase developmental competence via preventing ROS accumulation.

HTF medium triggered excessive production/accumulation of ROS when compared to KSOM. Embryos microinjected with recBCL-XL (ΔTM) protein and maintained in HTF medium had significantly reduced ROS levels (Figure 3B). Supplementing HTF medium with BCL-X derived BH4 domain TAT-synthetic peptide was sufficient to maintain a physiological ROS profile (Figure S5). However, developmental competence of embryos maintained in medium supplemented with BH4 peptide was not restored. These results indicate that the BH4 domain can alleviate excessive ROS, but only full length BCL-X protein, albeit lacking the transmembrane region (BCL-XL(ΔTM)), is capable of effectively restoring the developmental quality of embryos.

Previous work in bovine embryos identified the adaptor protein p66SHC as a mediator of permanent embryo arrest. Developmentally compromised bovine embryos have been reported to exhibit higher p66SHC expression accompanied by elevated ROS levels, and knockdown of *p66Shc* significantly reduced the occurrence of permanent embryo arrest in the bovine model [30], [31]. Immunocytochemistry revealed that recBCL-XL (ΔTM) microinjection decreased expression of both total and activated (pSer-36) p66SHC (Figure 3C) protein. As the P-p66SHC isoform has been shown to enter mitochondria and contribute to hydrogen peroxide release into the cytosol, decreased expression of p66SHC/Ser-36, particularly in the mitochondria, was accompanied by lower ROS levels in recBCL-XL (ΔTM) microinjected embryos.

**Figure 4:**
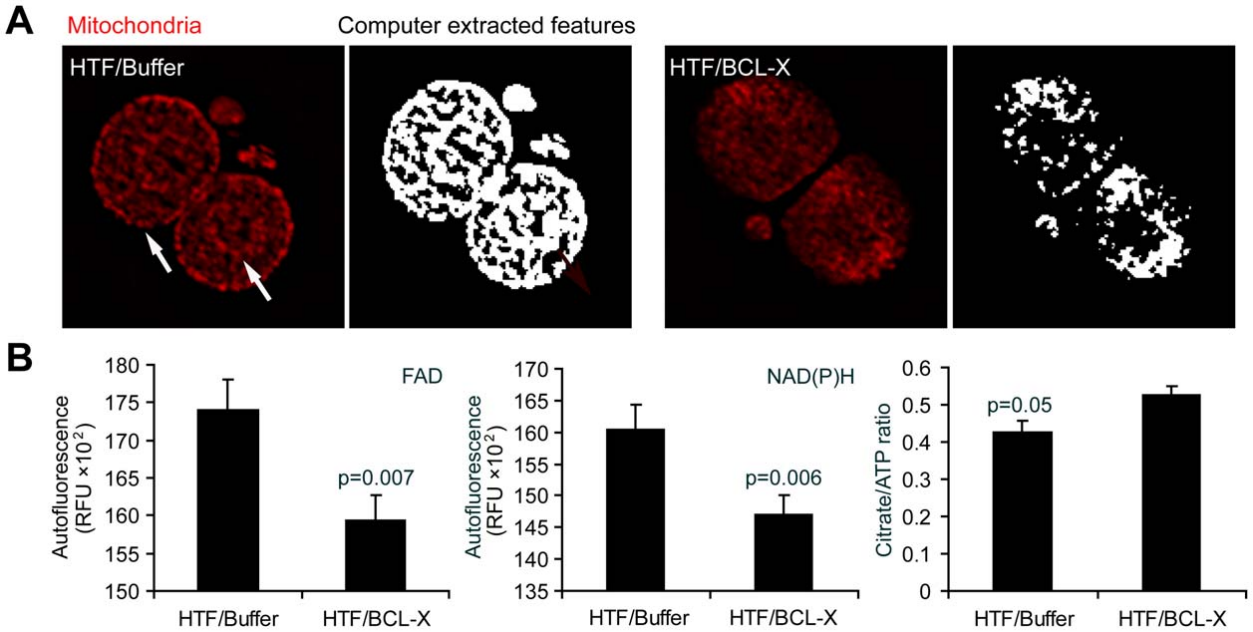
Impact of recBCL-XL (ΔTM) microinjection of embryo metabolism and mitochondrial distribution at 2-cell stage. (A) Mitochondrial distribution (Mitotracker Red) at 2-cell stage was evaluated by computerized image analysis approach (extracted features; see Text S1) and compared among cultured conditions. RecBCL-XL (ΔTM) protein maintained diffuse mitochondrial pattern (n = 30 embryos), while buffer (n = 30; similar to HTF culture alone), caused preferential clustering of these organelles to subcortical and perinuclear regions (arrows) of 2-cell embryos maintained in culture for 24 hours. (B) Microinjection of recBCL-XL (ΔTM) protein stabilized redox state of 2-cell stage embryos reduced (NAD(P)H and oxidized FAD autofluorescence signal expressed in RFU; n = 15 embryos per condition) and improved Krebs cycle outcome (Citrate/ATP ratio n = 15 embryos per condition). Student's t-test was used for statistical analysis. Bars indicate mean ± s.e.m.

Suboptimal culture conditions may result in both excessive ROS production and the alteration of embryo metabolism. BCL-X, in addition to its anti-apoptotic role, has also been implicated in mitochondrial biogenesis [32] and the regulation of mitochondrial metabolism [33]. Therefore, we next explored whether mitochondrial distribution, an indicator of embryo health [34], [35], can be altered by culture conditions. As anticipated, embryos cultured in HTF medium exhibit altered sub-cellular distribution of mitochondria in comparison to KSOM (Figure S6). Furthermore, this distribution was corrected by recBCL-XL (ΔTM) microinjection (Figure 4A), indicating that this Bcl-2 family member is capable of maintaining the correct cellular mitochondrial network in developing embryos.

We have also explored mitochondrial copy number (mtDNA content), mitochondrial activity (Mitotracker intensity) as well as mitochondrial membrane potential (JC-1 ratio). However, none of these parameters was affected by culture conditions or by BCL-X microinjection (data not shown). Finally, we investigated if recBCL-XL (ΔTM) microinjection can affect the metabolism of preimplantation embryos. Early embryos are not capable of glycolysis and rely on oxidative phosphorylation to sustain their energy demands. With development, they progressively gain the ability to utilize glucose [36]. Autofluorescent signals, reflecting cellular content of reduced NAD(P)H and oxidized FAD were significantly decreased in embryos upon microinjection of recBCL-XL(ΔTM) protein with concomitant increase in the citrate/ATP ratio (Figure 4B). Thus, BCL-X at the 2-cell stage modulates mitochondrial output, with outcomes indicating more efficient Krebs cycle metabolism and carbohydrate utilization.

### Expression of *BCL-X* in human oocytes

**Figure 5:**
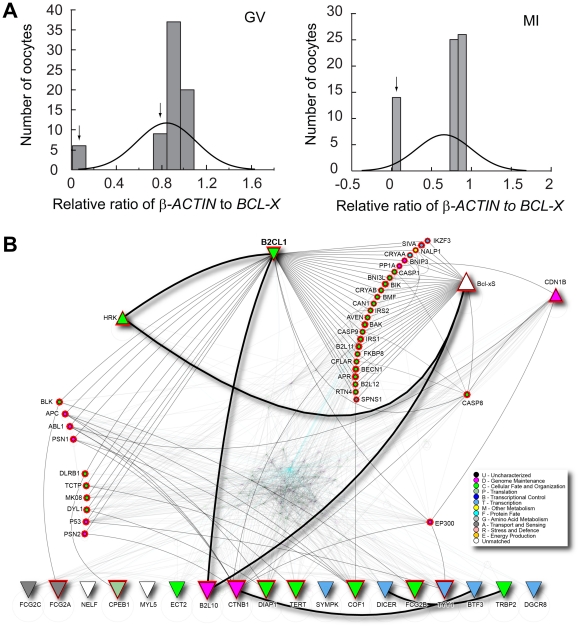
Expression of *BCL-X* in human oocytes. (A) Distribution of human oocytes obtained form 43 patients based on their *BCL-X* expression in either germinal vesicle stage (GV - left) or meiosis I stage (MI - right). Arrows point to groups of oocytes with insufficient endowment of *BCL-X* transcript. (B) Visualization of protein interaction network that connects BCL-X (BC2L1) with other targets known to be deregulated in arrested human embryos. Node shape, represented by triangles, indicates trends of expression. Shape of triangles pointing up corresponds to genes up-regulated and triangles pointing down correspond to genes down-regulated in arrested human embryos; circles represent direct interacting partners that link BCL-X (BCL2L1) to up- and down-regulated targets. Red highlight on nodes represents the set of cross-linked proteins. Node color is based on gene ontology as per legend. To reduce network complexity, all other nodes and edges are made partially transparent.

While experiments described above dealt with *in vitro* induced phenotype, they have clinical relevance to human IVF, where embryos are always maintained in culture for at least 3 days. In addition, it is also possible that some oocytes may lack sufficient storage of maternally derived *Bcl-x* gene products. In order to determine if variability in the endowment of maternally accumulated *BCL-X* transcripts could contribute to poor quality of human embryos in patients undergoing IVF, we analyzed its expression in human oocytes. As we have previously observed that *Bcl-x* transcript expression peaks in germinal vesicle (GV) stage of mouse oocytes, followed by dramatic decline in metaphase II stage [23], we used only human GV and metaphase I (MI) arrested oocytes. The GV and MI oocytes were analyzed separately. Within the cohort of oocytes obtained from patients undergoing infertility treatment, 6/72 GV and 14/65 MI oocytes failed to express detectable levels of *BCL-X* transcripts. An additional sub-group of oocytes (8 at GV stage) expressed reduced levels (less than mean) of *BCL-X* transcripts (Figure 5A). These data indicate that ∼20% of human growing oocytes obtained from infertile patients either completely lack or possess diminished endowment of *BCL-X* transcripts.

We next analyzed a possible link between BCL-X and genes known to be significantly deregulated in human arrested or fragmented embryos by considering known physical protein-protein interactions from the I2D database. These targets included genes ZAR1, YBX2, SYMPK, CPEB1, TARBP2, DICER1, DGCR8, MYLC2, ECT2, DIAPH1, CFL1, NELF, BTF3, IGFR2, YY1, TERT, DNMT3B, CTNNB1, HRK, BCL-XS, BCL2L10, P27KIP1 [23], [24], [37], [38], [39]. The resulting protein interaction network comprises 1,810 proteins and 20,562 interactions. Thick edges represent direct interactions among 22 up- and down-regulated genes/proteins. Thin, light grey edges link BCL-X (BCL2L1) with the up- and down-regulated genes, via 25 additional protein partners (small circles). On the left side are proteins that mostly link to down-regulated genes. EP300 is linked to both up- and down-regulated targets, while CASP8 is most linked to up-regulated genes (Figure 5B). This network highlights how lack of BCL-X may be connected with targets known to be differentially expressed in arrested embryos.

## Discussion

Enabled by the microfabricated embryo holding devices and vision-position based control of multiple motion control devices, the robotic mouse embryo injection system is capable of fast immobilization, switching, orientation, and injection of mouse embryos. The automated system requires minimal human involvement (i.e., a few computer mouse clicks), and is therefore, independent of operator skills, enabling the acquisition of large-scale molecule testing data with a high reproducibility. The robotic system performed microinjection of mouse zygotes at a speed of 12 zygotes/min, six times the speed of manual injection (∼2 zygotes/min). High accuracy and consistency of the robotic system produced lower cell lysis rates and higher blastocyst formation rates than proficient microinjection technicians. Except the micro devices for immobilizing embryos and the motorized rotational stage for orienting cells, the automated system contains no other custom developed components and presents no difference in hardware compared to a conventional microinjection system, which is an advantage promising its use in biology laboratories and mouse core facilities. The robotic cell manipulation technology presented here can also be adapted with further technological modifications and applied to other cell micromanipulation tasks, for instance, intracytoplasmic sperm injection (ICSI) and nuclear transfer procedures.

Our experimental results suggest that embryo arrest caused by suboptimal culture conditions is mostly driven by inefficient metabolism, in part due to decreased *Bcl-x* expression triggered by as yet unknown mechanisms. However, this altered cellular milieu can be restored by the microinjection of recombinant BCL-X protein, which repairs mitochondrial bioenergetics, prevents ROS accumulation, and facilitates the development of preimplantation mouse embryos. These effects start to become apparent at the 2-cell stage, and the consequences are still evident in the blastocyst stage (higher cell number and lower cell death rate; Figure 3A). While *Bcl-x* deletion causes midgestational embryonic lethality mostly due to defects in neuronal and hematopoietic lineages [40], its haplo-insufficiency triggers ovarian follicle loss, which became obvious with aging [41]. However, deletion of *Bcl-x* in follicles (oocyte and granulosa cells) did not compromise ovarian reserve in young age, but did result in decreased fertility of females [25].

We have previously shown that *Bcl-x* is maternally deposited into the oocytes, but becomes upregulated, likely from embryonic genome, at the late 1-cell stage [25]. In addition, some human as well as murine fragmenting early embryos alter splicing of *Bcl-x* gene, producing pro-apoptotic *Bcl-xS* isoform [24], [25]. We have recently shown that blastocysts do not require *Bcl-xL* for their survival, as downregulation of this isoform at the morula stage did not affect blastocyst quality. However, downregulation of *Bcl-xL* with concomitant induction of *Bcl-xS* had detrimental developmental consequences, as these blastocysts contain fewer cells and suffer from increased oxidative stress [42]. Data from the current study, however, point to a dependency on BCL-X during the critical window of transition from maternal to embryonic control, at the 2-cell stage.

The mechanism by which BCL-X promotes survival of 2-cell embryos does not appear to involve suppression of apoptosis, but rather points to the regulation of mitochondrial metabolism. These findings are consistent with previously published results in yeast and mammalian cell lines, which proposed that BCL-X can regulate a metabolic switch from glycolysis to oxidative phosphorylation [33], and can also maintain metabolite passage and activity of VDAC under conditions of stress [43], [44]. BCL-X and other BCL-2 family members had also been shown to dynamically remodel the mitochondrial network (e.g., fission and fusion; reviewed in [45]). Thus, changes in mitochondrial distribution without the effect on mitochondrial DNA copy number observed in recBCL-XL microinjected embryos, are not surprising.

Intriguing is, however, the connection between phosphorylation of p66SHC and BCL-X protein levels. It is presently unknown how BCL-X influences the phosphorylation of p66SHC, besides regulating ROS levels. Suppression of apoptosis by BCL-2 and BCL-XL can be attributed to protection against ROS and/or a shift of the cellular redox potential to a more reduced state [46]. Paradoxically, an elevated release of hydrogen peroxide was observed from BCL-XL overexpressing mitochondria, which led to an enhanced cellular antioxidant defense and superior protection against death [47]. It is also possible that dual crosstalk exists between BCL-X and p66SHC as previous work revealed that ablation of p66SHC increased expression of BCL-XL [48]. However, our data point to an additional role of BCL-X besides ROS safeguarding. While suboptimal culture clearly triggered oxidative stress, it is unlikely that ROS is the sole reason behind early embryo arrest, as BH4 peptide could alleviate the ROS accumulation, but could not support further embryo development. Likely, a combined role in mitochondrial metabolism with physiological ROS maintenance facilitates the viability of early embryos.

We have also attempted to explore if microinjection of recBCL-XL protein could have a clinical relevance. Expression screen of oocytes from infertile patients revealed variability in the *BCL-X* endowment. Within the cohort of freshly collected immature human oocytes, one-fifth either lacked or expressed lower amounts of *BCL-X* transcripts. However, the level of *BCL-X* did not correlate with clinical parameters such as patient infertility diagnosis, maternal age, stimulation protocol as well as pregnancy outcome (data not shown). We observed that oocytes from the same patient would be variably affected, indicating that not all oocytes are created equal and likely would not equally well support preimplantation embryo development. We speculate that embryos conceived from oocytes lacking BCL-X would be incapable of progression through preimplantation development and would arrest during *in vitro* culture. As we did not assess expression of *BCL-X* in the transferred embryos, this may explain the lack of *BCL-X* correlation with pregnancy outcome. Nonetheless, microinjection of recBCL-XL could have potential use during IVF treatment, particularly for cases where patients experience repeated IVF failure due to poor embryo quality.

Microinjection of recombinant mitochondrial proteins is capable of improving the developmental competence of embryos without genetic alterations in the offspring, a problematic factor of ooplasmic transfer. In contrast to mitochondrial heteroplasmy that results from ooplasmic transfer, the recombinant form of the BCL-X protein has a terminal half-life. Therefore, its addition to the embryos is aimed at providing transient support during the time that embryos are most susceptible to demise without resulting in permanent genetic modifications of the offspring. Further screening of additional protein targets using the automated microinjection technique could lead to the selection of the most efficacious proteins for improving embryo survival.

## Materials and Methods

### Ethics Statement

All mouse experiments were performed in accordance with Canadian Council on Animal Care (CCAC) guidelines for Use of Animals in Research and Laboratory Animal Care under protocols (permit or protocol #:AUP0015) approved by the animal care committees at Mount Sinai Hospital (MSH), Toronto and Toronto Centre for Phenogenomics (TCP). Single immature human oocytes at germinal vesicle or metaphase I stage were donated to research after obtaining patient consent approval in writing, which was approved by the Research Ethics Board at Mount Sinai Hospital, Toronto.

### Mouse Embryo Collection and Culture

The collection and culture conditions of mouse embryos are described in the Supporting Methods (Text S1).

### Manual and Robotic Microinjection

The workstation for manual microinjection consisted of an inverted microscope equipped with differential interference contrast (DIC) optics (Leica Microsystems, Wetzlar, Germany). Microinjection pipettes were backloaded and connected to a microinjector (FemtoJet; Eppendorf, Hamburg Germany). Holding pipettes (100 µm O.D., 30 µm I.D.) were prepared on a microforge (DeFonbrune) from 1.0 mm O.D.×0.75 mm I.D. borosilicate glass capillaries (FHC 27-30-0) and connected to a manually controlled oil-based holding syringe system (Narishige, Japan). Mouse zygotes with visible pronuclei were selected for microinjection. All the zygotes were injected within the time window of 1–3 hr post-collection.

Detailed description of the robotic microinjection system design and operation is provided in the Supporting Methods (Text S1) and Videos.

### Measurement of Reactive Oxygen Species (ROS) Content

In the experiments, ROS content of the injected and un-injected embryos was measured at the 2-cell stage. The level of ROS content was quantified using the dichlorodihydrofluorescein diacetate (DCHFDA, Molecular Probes, Invitrogen, Carlsbad, CA, USA) method as previously described [49]. Live imaging and quantitation were conducted on a deconvolution microscope (Olympus IX70, Applied Precision Inc. Issaquah, WA, USA) using an image analysis program (SoftwoRx, Applied Precision Inc., Issaquah, WA, USA).

### Immunocytochemistry for pSHC and BCL-X

Embryos, fixed with 10% formalin, were incubated overnight with appropriate primary antibody, rabbit anti-SHC (BD Transduction laboratories), mouse anti-SHC/phospho S36 antibody (Abcam) or rabbit anti mouse BCL-X (Santa Cruz, CA). After washing, embryos were incubated with appropriate secondary antibodies. Following a 15 minute counterstain with DAPI, embryos were mounted and imaged on a deconvolution microscope (Olympus IX70; Applied Precision Inc., Issaquah, WA, USA), and relative fluorescence in each image was quantitated as described above.

### Metabolic Assays and Mitochondrial Labeling (Mitotracker)

Microanalytical metabolic assay for ATP and citrate levels were performed as previously described [50]. Live embryos were stained with Mitotracker Red (Molecular Probes, Invitrogen, Carlsbad, CA, USA) at a final concentration of 200 µM. Embryos were then imaged on a deconvolution microscope (Olympus IX70, Applied Precision Inc., Issaquah, WA, USA) under the TRITC filter. NAD(P)H and FAD autofluorescence of the embryos were also imaged under DAPI and FITC filters, respectively as previously described [51]. Quantitation of the mitochondrial DNA copy number and mitochondrial membrane potential (JC-1, Molecular probes, Invitrogen, Carlsbad, CA, USA) labeling was performed as previously described [34] and distribution was evaluated as outlined in Supporting Methods (Text S1).

### Network Analysis and Visualization

Genes reported to be differentially expressed in human arrested or fragmented embryos, were chosen based on previous publications [23], [24], [37], [38], [39]. These gene targets were mapped to proteins and used to assess connectivity to *BCL-X* using the known, physical protein-protein interactions. Network was generated by querying I2D database Version 1.95 [52], [53]. Network visualization was performed in NAViGaTOR 2.2 ([54], [55]).

### Statistical Analysis

Data were presented as mean ± s.e.m. and analyzed using either student's t-test, Mann-Whitney U-test, or Kruskal Wallis test followed by Dunn's post test (SigmaStat 3.5, Systat Software Inc.), as appropriate. For patient data analysis, Pearson correlation was used for maternal age and Anova on Ranks for patient diagnosis, hormonal stimulation and pregnancy outcome. All the statistical analyses were performed using SigmaStat 3.5 (Systat Software Inc.).

## Supporting Information

Figure S1 **Zygote immobilization using a glass-based cell holding device.** (**A**) A completed glass cell holding device. (**B**) A zoomed-in picture of the through holes. (**C**) A 5×5 array of immobilized mouse zygotes using the cell holding device.
(TIF)

Figure S2 **Overall flow of microrobotic mouse embryo injection.** (**A**) Contact between micropipette tip and cell holding cavity is detected using a vision-based algorithm [15]. (**B**) The micropipette tip is elevated to a *home* position *H*, and the first embryo is brought into the field of view, recognized and centered. If the polar body faces the penetration site, the embryo is properly rotated through automatic orientation control. (**C**) Micropipette is moved to a switch point, *S*. (**D**) The micropipette penetrates the embryo and deposits materials to the target destination. (**E**) The micropipette is retracted out of the embryo. (**F**) Micropipette is moved to the *home* position. Simultaneously, the next embryo is brought into the field of view. This injection process is repeated until all the embryos in the batch are injected.
(TIF)

Figure S3 **Mouse zygote orientation.** (**A**) Side view and (**B**) top view of the zygote and injection micropipette before orientation. When the polar body appears in the space of quadrant II, there are risks of either direct polar body penetration or large stress induced polar body damage. The desired target orientation is either 12 o'clock or 6 o'clock. (**C**) Top view of the embryo after orientation. Polar body
(TIF)

Figure S4 **Impact of injection modes of delivery (manual vs. automated injection) on rates of blastocyst formation and embryo quality.** Both modes of delivery significantly improved developmental potential of embryos injected with recBCL-XL (ΔTM) protein (manual injection: n = 107 for buffer and n = 122 for protein; automated injection: n = 307 for buffer and n = 302 for protein). No significant difference (p = 0.359 for buffer injection; p = 0.762 for protein injection) was found between rates of blastocyst formation if protein was delivered into either cytoplasm or pronucleus. Microinjection of recBCL-XL (ΔTM) protein also significantly enhanced the embryo quality (manual injection: n = 39 for buffer and n = 65 for protein; automated injection: n = 32 for buffer and n = 44 for protein). Bars indicate mean ± s.e.m. Student's t-test was used for pairwise comparison.
(TIF)

Figure S5 **Impact of culture medium on reactive oxygen species (ROS) levels.** Assessment of the relative amounts of ROS measured by DCHFDA probe in 2-cell embryos cultured for 24 hours in KSOM (n = 15), HTF (n = 17), HTF with 15 ng/µl of BH4 peptide (n = 15). Bars indicate mean ± s.e.m. Kruskal Wallis test followed by Dunn's post test was used for statistical analysis.
(TIF)

Figure S6 **Computationally quantitated mitochondrial distributions at 2-cell stage in (A) un-injected and (B) injected embryos.** Using Euler number computation, mitochondrial distribution in un-injected embryos is significantly altered by culture medium (n = 19 for HTF; n = 20 for KSOM), which can be corrected by microinjection of recBCL-XL (ΔTM) protein (n = 22 for each condition). Bars indicate mean ± s.e.m. Mann-Whitney U-test was used for pairwise comparison.
(TIF)

Video S1 **Vacuum-based cell immobilization using a microfabricated glass device.**
(MOV)

Video S2 **Automated microinjection of mouse zygotes using a robotic cell injection system.**
(MOV)

Text S1 **Supporting methods.**
(DOC)
