# Supplementary figures and images for: Automated Microinjection of Recombinant BCL-X into Mouse Zygotes Enhances Embryo Development
Source: PLoS One. 2011 Jul 20;6(7):e21687. doi: 10.1371/journal.pone.0021687 (PMC3140481; doi:10.1371/journal.pone.0021687)

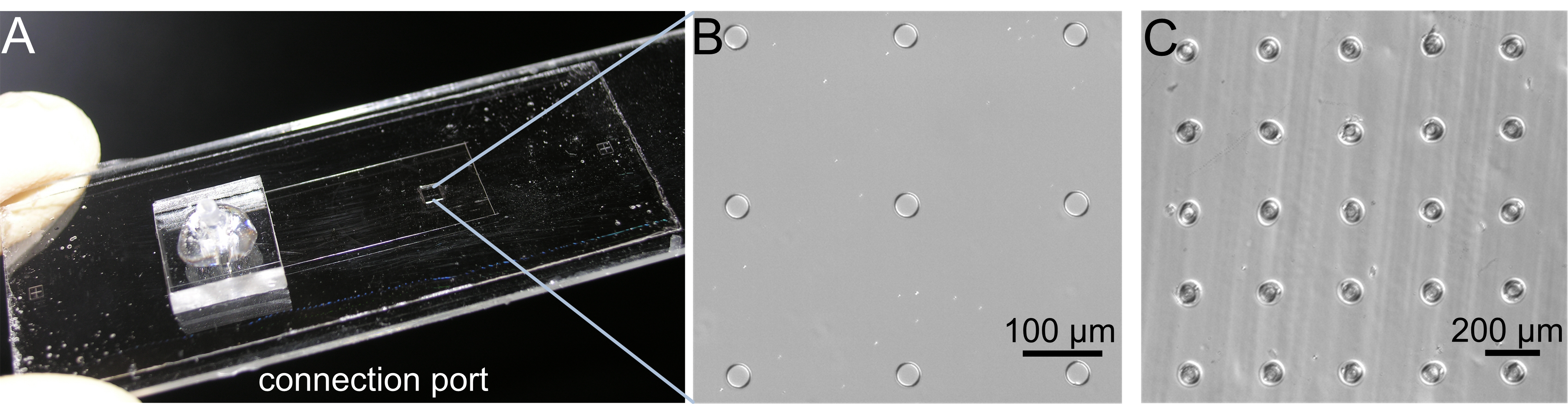

Supplement: Figure S1 — Zygote immobilization using a glass-based cell holding device. (A) A completed glass cell holding device. (B) A zoomed-in picture of the through holes. (C) A 5×5 array of immobilized mouse zygotes using the cell holding device. (TIF) [file pone.0021687.s001.tif]

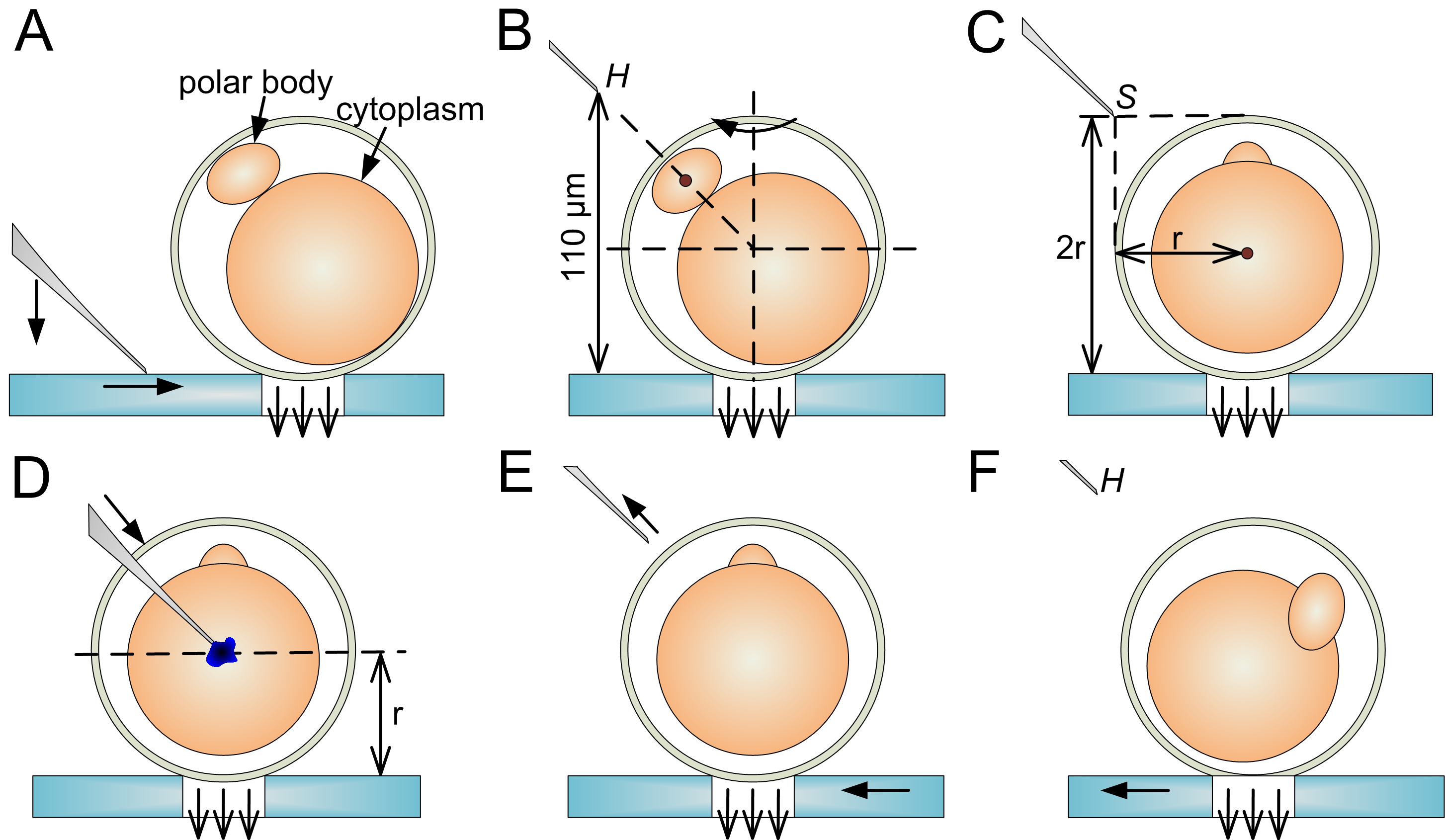

Supplement: Figure S2 — Overall flow of microrobotic mouse embryo injection. (A) Contact between micropipette tip and cell holding cavity is detected using a vision-based algorithm [15]. (B) The micropipette tip is elevated to a home position H, and the first embryo is brought into the field of view, recognized and centered. If the polar body faces the penetration site, the embryo is properly rotated through automatic orientation control. (C) Micropipette is moved to a switch point, S. (D) The micropipette penetrates the embryo and deposits materials to the target destination. (E) The micropipette is retracted out of the embryo. (F) Micropipette is moved to the home position. Simultaneously, the next embryo is brought into the field of view. This injection process is repeated until all the embryos in the batch are injected. (TIF) [file pone.0021687.s002.tif]

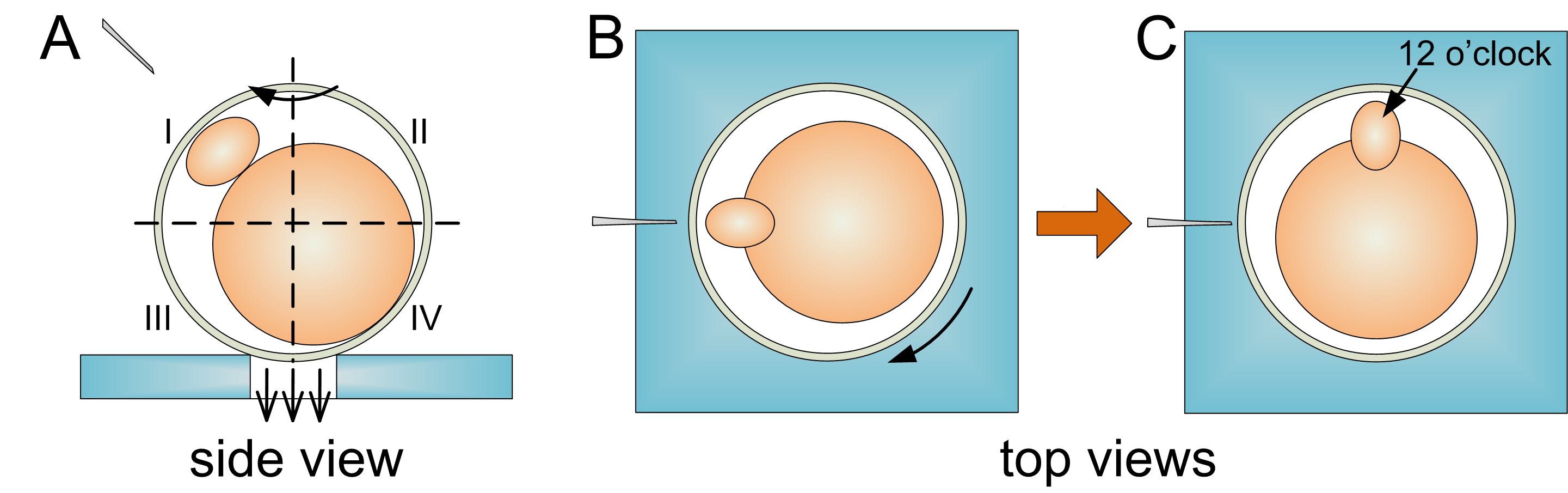

Supplement: Figure S3 — Mouse zygote orientation. (A) Side view and (B) top view of the zygote and injection micropipette before orientation. When the polar body appears in the space of quadrant II, there are risks of either direct polar body penetration or large stress induced polar body damage. The desired target orientation is either 12 o'clock or 6 o'clock. (C) Top view of the embryo after orientation. Polar body (TIF) [file pone.0021687.s003.tif]

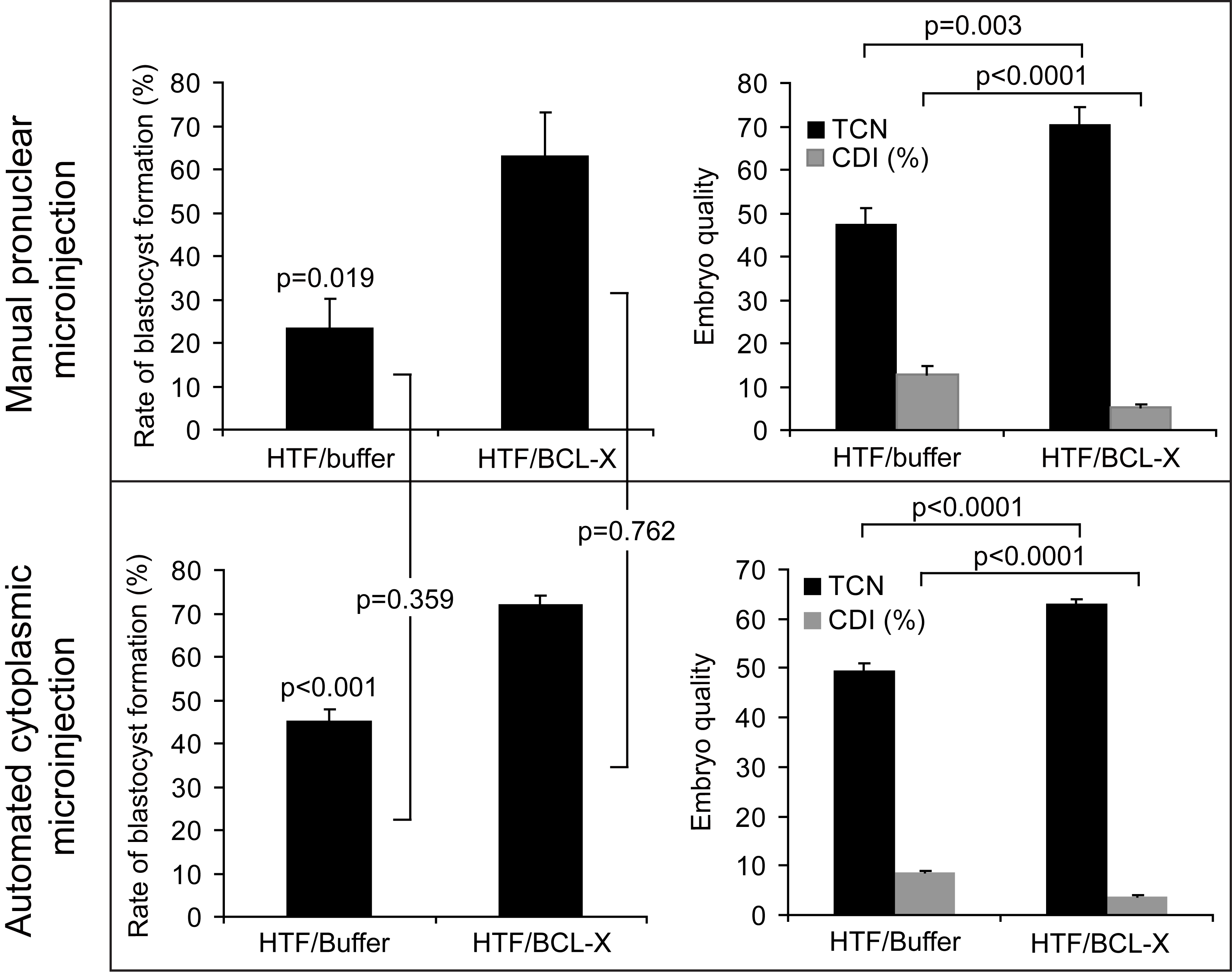

Supplement: Figure S4 — Impact of injection modes of delivery (manual vs. automated injection) on rates of blastocyst formation and embryo quality. Both modes of delivery significantly improved developmental potential of embryos injected with recBCL-XL (ΔTM) protein (manual injection: n = 107 for buffer and n = 122 for protein; automated injection: n = 307 for buffer and n = 302 for protein). No significant difference (p = 0.359 for buffer injection; p = 0.762 for protein injection) was found between rates of blastocyst formation if protein was delivered into either cytoplasm or pronucleus. Microinjection of recBCL-XL (ΔTM) protein also significantly enhanced the embryo quality (manual injection: n = 39 for buffer and n = 65 for protein; automated injection: n = 32 for buffer and n = 44 for protein). Bars indicate mean ± s.e.m. Student's t-test was used for pairwise comparison. (TIF) [file pone.0021687.s004.tif]

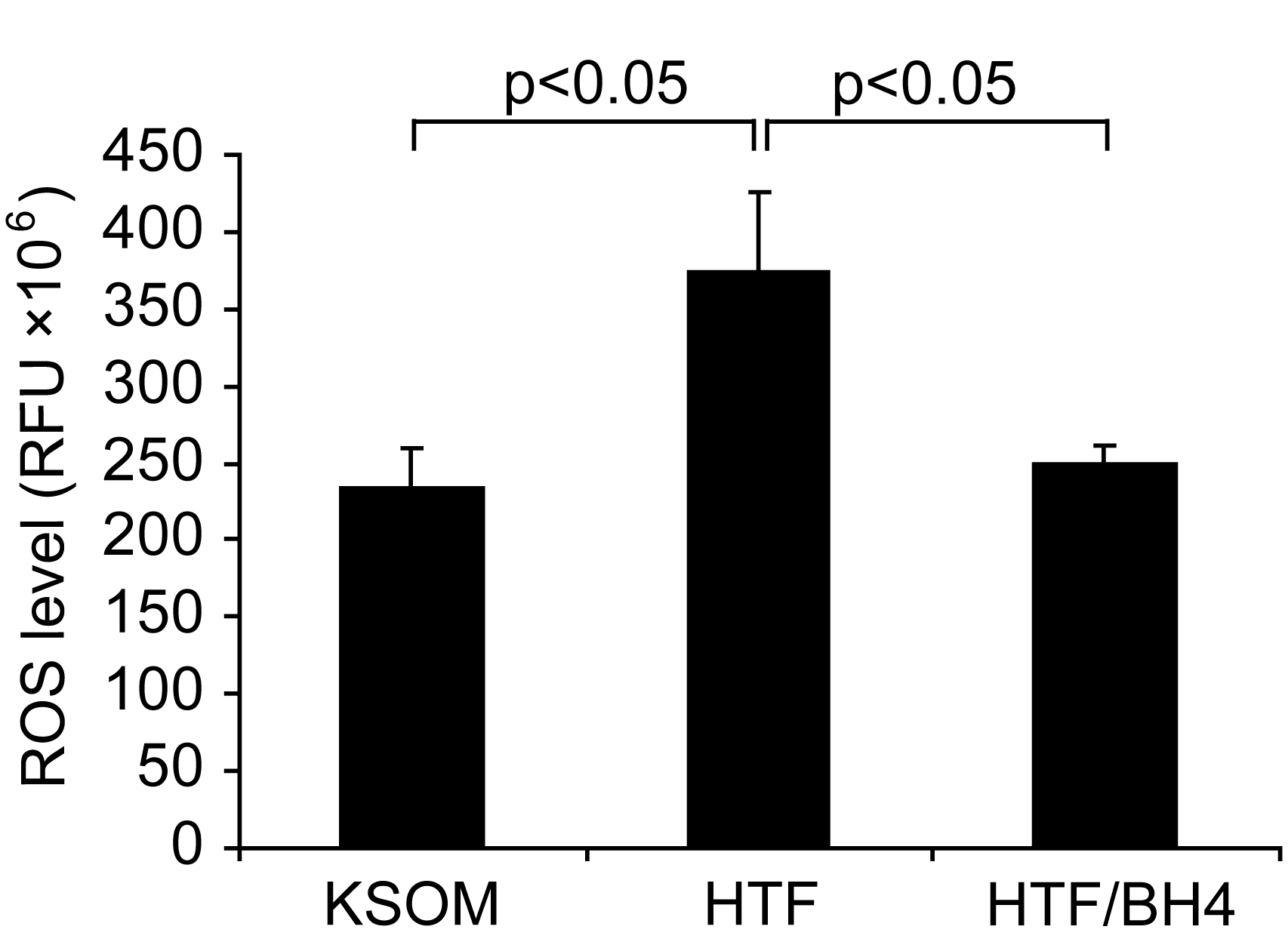

Supplement: Figure S5 — Impact of culture medium on reactive oxygen species (ROS) levels. Assessment of the relative amounts of ROS measured by DCHFDA probe in 2-cell embryos cultured for 24 hours in KSOM (n = 15), HTF (n = 17), HTF with 15 ng/µl of BH4 peptide (n = 15). Bars indicate mean ± s.e.m. Kruskal Wallis test followed by Dunn's post test was used for statistical analysis. (TIF) [file pone.0021687.s005.tif]

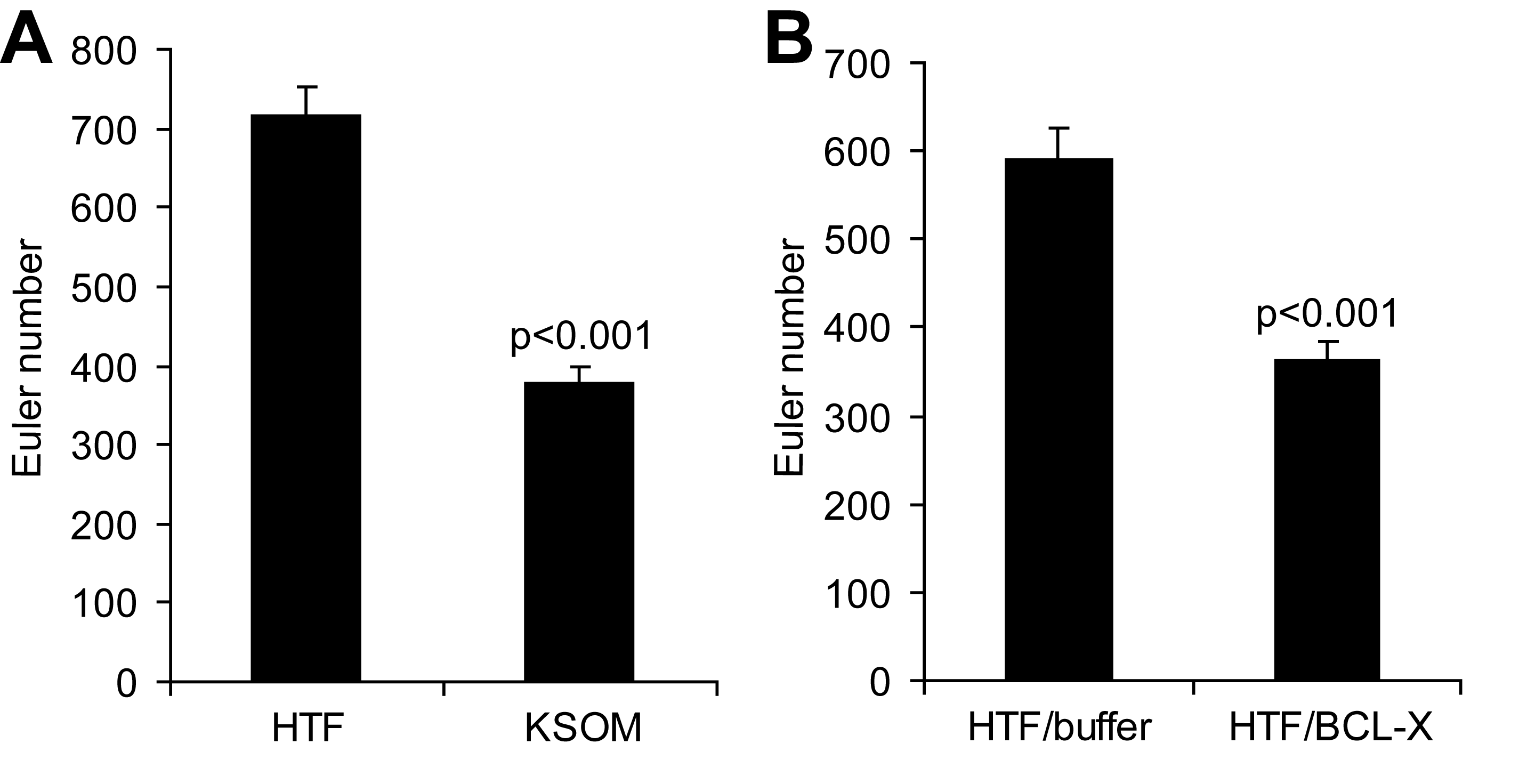

Supplement: Figure S6 — Computationally quantitated mitochondrial distributions at 2-cell stage in (A) un-injected and (B) injected embryos. Using Euler number computation, mitochondrial distribution in un-injected embryos is significantly altered by culture medium (n = 19 for HTF; n = 20 for KSOM), which can be corrected by microinjection of recBCL-XL (ΔTM) protein (n = 22 for each condition). Bars indicate mean ± s.e.m. Mann-Whitney U-test was used for pairwise comparison. (TIF) [file pone.0021687.s006.tif]
