# Supplementary material for: Automated Microinjection of Recombinant BCL-X into Mouse Zygotes Enhances Embryo Development
Source: PLoS One. 2011 Jul 20;6(7):e21687. doi: 10.1371/journal.pone.0021687 (PMC3140481; doi:10.1371/journal.pone.0021687)
Supplement: Text S1 — Supporting methods. (DOC) [file pone.0021687.s009.doc]

**Supporting Methods**

# Automated Microinjection of Recombinant BCL-X into Mouse Zygotes Enhances Embryo Development

Xinyu Liu^1#^, Roxanne Fernandes^2#^, Marina Gertsenstein^2^, Alagammal Perumalsamy^2^, Ingrid Lai^2^, Maggie Chi^3^, Kelle H. Moley^3^, Ellen Greenblatt^4^, Igor Jurisica^5^, Robert F. Casper^2,4^, Yu Sun^1^*, and Andrea Jurisicova^2,4^*

**Animal husbandry and Culture Conditions**

Animals were maintained on 12 h light/dark cycle and provided with food and water *ad libitum* in open cages (MSH) or individually ventilated units (TCP). Female ICR mice (Harlan) at 6 weeks of age were stimulated with 5 IU of pregnant mare serum gonadotropin (PMSG) (National Hormone and Peptide Program, NIDDK, Bethesda MD) followed 48 hours later by 5 IU of human chorionic gonadotropin (hCG) (Calbiochem, Gibbstown, NJ, USA) by intraperitoneal injection and mated overnight with ICR males. Embryos were recovered from the oviducts at 0.5 day post coitum (dpc) at the zygote stage. Ampullae were opened in 0.3 mg/ml hyaluronidase (Sigma-Aldrich, St. Louis, MO, USA) in modified HTF (mHTF; Irvine Scientific, Irvine, CA, USA) to release cumulus masses.

Before microinjection experiments, 20 μl droplets of either KSOM supplemented with amino acids (KSOM; Millipore,  Billerica, MA, USA) or HTF medium Irvine Scientific, Irvine, CA, USA) supplemented with 0.1% BSA (Sigma-Aldrich, St. Louis, MO, USA.) were set up in 35 mm Petri dishes, covered with mineral oil, and pre-equilibrated overnight in a humidified 37°C incubator with 5% CO_2_. Microinjection of mouse zygotes was conducted in mHTF. After injection with either recBcl-xL (ΔTM) protein (1.5 μg/μl) diluted in EmbryoMax Injection buffer (Millipore, Billerica, MA, USA) or buffer alone, embryos were cultured in appropriate medium with 10-20 embryos per group. For some experiments, TAT-BH4 peptide (Calbiochem, Gibbstown, NJ, USA) was supplemented in HTF culture medium (15 ng/μl). In addition, uninjected embryos were also cultured in HTF and KSOM droplets as control groups. Developmental progression was recorded daily. At day 4.5 (~96 hours in culture) embryos were fixed and total cell number and cell death index were determined as previously described [1].

**Mitochondrial distribution analysis**

Computer-based morphometry was used to quantitate differences in mitochondrial distribution, as previously described [2]. Briefly, we identified regions of the stained mitochondria using Mitotracker red staining (Figure 4A), and quantitated mitochondrial distribution by calculating an Euler number. The Euler number measures the topology of an image and is defined as the number of objects in the image minus the number of holes in those objects. A lower Euler number indicates more clustered mitochondria, while higher number is indication of higher fragmentation of mitochondria.

**Real time RT-PCR**

Obtained single human oocytes, free of cumulus cells were loaded into guanidine iso-thio cyanate (GITC) solution and total nucleic acid was precipitated as previously described [3,4]. Upon treatment with DNase (Sigma-Aldrich, St.Louis, MO, USA), reverse transcription was performed on whole sample using Fermentas Revert Aid kit (Fermentas, Burlington, Ontario, Canada) and oligo-dT primers. 1/40 of the cDNA product was used in a real time PCR reaction using LightCycler® 480 SYBR Green I Master (Roche Applied Science, Indianapolis, IN, USA) and LightCycler® 480 (Roche, Mannheim, Germany). Primer sequences were located in the 3’UTR corresponding to *β-ACTIN* (Forward: 5’-CACAGGGGAGGTGATAGCAT-3’; Reverse: 5’-CACGAAGGCTCATCATTCAA-3’) or *BCL-X* (Forward: 5’-GAGCTGTTTATGGCCTCAGC-3’ Reverse: 5’-GCTCCCATAGCTGTTCCTGA-3’) and the samples were run simultaneously in duplicates for each gene. Amplification profile included a pre-incubation step at 95 °C for 5 minutes, followed by denaturation at 95 °C for 20 s, annealing at 62 °C for 15 s and extension at 72 °C for 15 s. Initially, all oocyte were pre-screened for *β-ACTIN* expression and the samples that had a crossing point (C_P_) value less than 35 were used for further analysis. The target gene concentration for the oocyte samples was extrapolated utilizing the standard curve for *BCL-XL* and the data were expressed as relative ratio C_P_ value of *β-ACTIN*.

**Robotic Mouse Embryo Injection System**

We partially described the technical development of the robotic mouse embryo injection system previously [5,6]. To make this paper self-contained and facilitate the discussion of the testing results of system performance, we briefly summarize the key features of the robotic system as follows.

**Glass cell holding device for cell immobilization:** Vacuum-based glass cell holding devices (Figure 1A and Figure S1A) were constructed via standard microfabrication for rapidly immobilizing an array of mouse embryos [5]. Briefly, micrometer-sized through-holes (37 μm±0.5 μm in diameter) are formed on a standard cover slip (~170 μm thick) using two-side hydrofluoric acid (HF) wet etching. The device consists of a top glass layer with an array of through-holes, a bottom glass layer, and a PDMS spacer for forming a vacuum chamber. Devices with arrays of 3×3 and 5×5 through-holes were used for immobilizing mouse zygotes (~98 µm). Low pressures of 1.6 kPa-2.2 kPa were experimentally determined to be effective for holding the cells in place with sufficient forces during micropipette penetration. The complete cell immobilization process including the removal of extra cells typically takes 31 s for devices with an array of 5×5 through holes.

**Injection micropipette fabrication and volume control:** The injection micropipette is pulled using a programmable micropipette puller (P-97, Sutter), and the tip of pulled micropipette is then abraded using a micropipette beveler (BV-10, Sutter) to form a sharp tip and a 1.2 μm opening (inlet picture in Figure 1E). This micropipette fabrication process is highly repeatable, providing precisely controlled shape and size of the micropipette tip.

The deposition volume as a function of the pressure level and deposition time was accurately calibrated by measuring the size of spherical droplet blown out of the micropipette tip under an inverted microscope. Figure 1E shows a calibration data example of the deposition volume using deionized water. The resolution of material deposition volume was 1fL.

Micropipettes with an inner diameter (I.D.) of 1.2 µm and a sharp angle of 10º were used in experiments for evaluating the system performance. Due to protein viscosity, micropipettes with an I.D. of 3 µm and a sharp angle of 20º were used for injecting the BCL-XL (ΔTM) protein and TE buffer.

**Injection control sequence:** A batch of mouse embryos are transferred and immobilized on the embryo holding device placed on the motorized rotational stage. Control flow of the injection process, shown in Figure S2, starts with vision-based contact detection [7] to vertically align the injection micropipette tip and the bottom surface of the cell holding cavity. The injection pipette tip is then raised to a *home* position, *H* (Figure S2B).

Simultaneously, the first embryo is brought into the field of view. The cytoplasm center and the polar body center are identified visually by a human operator and input to the system through computer mouse clicking. The embryo is then brought to the center of the field of view by the X-Y translational stage. If the polar body faces the penetration site, it is rotated away via visually servoed embryo orientation control.

Once the embryo is oriented, if needed, the microrobot controls the micropipette to a switch point, *S*. After the switch point, the micropipette diagonally penetrates the embryo and deposits materials at the injection destination (i.e., cytoplasm center). Upon retracting out of the embryo (Figure S2E), the micropipette is moved back to the *home* position, *H*. The next embryo is then brought into the field of view (Figure S2F). This injection process is repeated until all the embryos in the batch are injected.

Throughout the process, motions of the X-Y stage and 3-DOF micro-robot are controlled via proportional-integral-derivative (PID) control law. Transformations among the multiple coordinate frames are achieved during the operation of the system without requiring an off-line process.

**Embryo structure identification:** The determination of the injection destination (cytoplasm center) and the polar body center for orientation control is conducted by a human operator and input to the robotic system via computer mouse clicking in the graphical user interface of the control software. The system remains this minimal human-machine intervention to guarantee reliability. When the polar body of an embryo is within the depth of field, the human operator directly selects image coordinates of the cytoplasm and polar body centers. When the polar body is not visible (out of focus), the embryo is focally scanned by the *z*-motor on the microscope. During this scanning process, the human operator identifies and selects the polar body center. The control software accepts the image coordinates of the cytoplasm and polar body centers from user input, which are subsequently converted into Cartesian frame coordinates via online coordinate transformations. In the experiments, we found that only 8.2% of the injected embryos appeared in the space of quadrant II (Figure S3A), which required automated re-orientation.

**Vision-based cell orientation control:** Since an embryo within a batch is rarely coincident along the rotational axis of the rotational stage, coupled translational motions during rotation cause the embryo to move out of the field of view. Thus, the system conducts 3-DOF cooperative control of the X-Y translational stage and the rotational stage to bring the embryo back into the field of view after orientation [8]. This control method permits high-speed orientation of cells.

Coordinate transformation between the frames of the rotational stage and X-Y translational stage is calibrated by image-based visual servoing [9] of the X-Y stage for always keeping the embryo inside the field of view during cell orientation. This calibration procedure is only required/conducted on the first embryo that requires re-orientation for an entire batch of immobilized embryos, since pitches (300μm) between adjacent embryos are accurately known.

**Supporting References**

1. Jurisicova A, Rogers I, Fasciani A, Casper RF, Varmuza S (1998) Effect of maternal age and conditions of fertilization on programmed cell death during murine preimplantation embryo development. Mol Hum Reprod 4: 139-145.

2. Acton BM, Jurisicova A, Jurisica I, Casper RF (2004) Alterations in mitochondrial membrane potential during preimplantation stages of mouse and human embryo development. Mol Hum Reprod 10: 23-32.

3. Jurisicova A, Antenos M, Varmuza S, Tilly JL, Casper RF (2003) Expression of apoptosis-related genes during human preimplantation embryo development: potential roles for the Harakiri gene product and Caspase-3 in blastomere fragmentation. Mol Hum Reprod 9: 133-141.

4. Jurisicova A, Latham KE, Casper RF, Varmuza SL (1998) Expression and regulation of genes associated with cell death during murine preimplantation embryo development. Mol Reprod Dev 51: 243-253.

5. Liu X, Sun Y (2009) Microfabricated glass devices for rapid single cell immobilization in mouse zygote microinjection. Biomed Microdevices 11: 1169-1174.

6. Liu X, Sun Y. Automated mouse embryo injection moves toward practical use. IEEE International Conference on Robotics and Automation (ICRA2009), Kobe, Japan, May 12-17, 2009.

7. Wang WH, Liu XY, Sun Y (2007) Contact detection in microrobotic manipulation. Int J Robot Res 26: 821-828.

8. Liu X, Lu Z, Sun Y (2011) Orientation Control of Biological Cells Under Inverted Microscopy. IEEE/ASME Trans Mechatronics DOI: 10.1109/TMECH.2010.2056380, 2011.

9. Hutchinson S, Hager GD, Corke PI (1996) A tutorial on visual servo control. IEEE Trans Robot Automat 12: 651-670.
